# Involvement of Exogenous *N*-Acyl-Homoserine Lactones in Spoilage Potential of *Pseudomonas fluorescens* Isolated From Refrigerated Turbot

**DOI:** 10.3389/fmicb.2019.02716

**Published:** 2019-11-29

**Authors:** Tingting Li, Dangfeng Wang, Likun Ren, Yongchao Mei, Ting Ding, Qiuying Li, Haitao Chen, Jianrong Li

**Affiliations:** ^1^Key Laboratory of Biotechnology and Bioresources Utilization, Ministry of Education, Dalian Minzu University, Dalian, China; ^2^College of Food Science and Technology, Bohai University, National and Local Joint Engineering Research Center of Storage, Processing and Safety Control Technology for Fresh Agricultural and Aquatic Products, Jinzhou, China; ^3^School of Food Science and Technology, Jiangnan University, Wuxi, China; ^4^Beijing Key Laboratory of Flavor Chemistry, Beijing Technology and Business University, Beijing, China

**Keywords:** quorum sensing, *Pseudomonas fluorescens*, spoilage potential, eavesdropping, molecular docking

## Abstract

Some bacteria can modulate their spoilage potential by responding to environmental signaling molecules via the quorum sensing (QS) system. However, the ability of *Pseudomonas fluorescens*, the specific spoilage organism (SSO) of turbot, to response to environmental signaling molecules remains unclear. This study investigated the effects of six synthetic *N*-acyl homoserine lactones (AHLs) on typical behaviors mediated by QS in *P. fluorescens*, such as biofilm formation and extracellular protease activity. Total volatile basic nitrogen (TVB-N) was used as a spoilage indicator to evaluate quality changes in AHL-treated turbot filets during storage. The results confirm the enhancing effect of environmental AHLs on QS-dependent factors of *P. fluorescens* and quality deterioration of turbot filets, with C_4_-HSL and C_14_-HSL being the most effective. Moreover, the content decrease of exogenous AHLs was also validated by gas chromatography–mass spectrometry analysis. Further, changes in *rhlR* transcription levels in *P. fluorescens* suggest that this bacterium can sense environmental AHLs. Finally, molecular docking analysis demonstrates the potential interactions of RhlR protein with various exogenous AHLs. These findings strongly implicate environmental AHLs in turbot spoilage caused by *P. fluorescens*, suggesting preservation of turbot should not exclusively consider the elimination of SSO-secreted AHLs.

## Introduction

Bacterial growth and metabolism results in aquatic food spoilage through protein degradation and formation of unacceptable volatile constituents, such as amines, alcohols, aldehydes, and sulfides (Gram and Dalgaard, 2002). Specific spoilage organisms (SSOs) play a crucial role in this process. Recent studies have indicated that bacterial spoilage may be regulated by a cell density-dependent communication mechanism called quorum sensing (QS), which is based on the self-secretion and detection of QS signaling molecules (Fuqua et al., 1994; Gui et al., 2017; Zhao et al., 2017). Bacteria trigger the expression of factors required for food spoilage (such as extracellular proteases, lipases, pectases, and cellulases) or pathogenesis (such as biofilm formation, motility alteration, hemolysin production, and pyocyanin generation) when the concentration of these signaling molecules reaches a certain threshold level (Bai and Rai, 2011). Interestingly, different microorganisms rely on different types of signaling molecules to regulate their QS systems (Papenfort and Bassler, 2016). *Pseudomonas aeruginosa* typically regulates collective behaviors via a wide range of signaling molecules such as 3-oxo-C_12_-HSL, C_4_-HSL, 2-heptyl-3-hydroxy-4-quinolone (PQS), 2-heptyl-4-hydroxyquinoline (HHQ), and 2-(2-hydroxyphenyl)-thiazole-4-carbaldehyde (IQS). Alternatively, *Vibrio harveyi* typically employs secreted harveyi autoinducer-1 (HAI-1), autoinducer-2 (AI-2), and cholerae autoinducer-1 (CAI-1) as signaling molecules (Galloway et al., 2011; Defoirdt, 2017). Recent studies have found that the above signaling molecules are often detected in contaminated aquatic products (Li et al., 2016; Zhao et al., 2017). Accordingly, additional work is still needed to explore the potential relationship between signaling molecule-based QS system and food spoilage.

*Pseudomonas fluorescens* is a gram-negative bacterium widely distributed in soil, plants, and the marine environment. Moreover, *P. fluorescens* possesses the characteristics of psychrotrophs, which are often associated with the spoilage of food stored at low temperatures, such as aquatic products and milk (Bandlitz and Berke, 2013; Stuknytė et al., 2016). Like most gram-negative bacteria, *N*-acyl-homoserine lactones (AHLs) have been detected in many foods spoiled by *P. fluorescens* (Liu et al., 2008; Bai and Rai, 2011). This suggests that the AHL-mediated QS system may be an important QS system in *P. fluorescens*, although it cannot be detected in all *P. fluorescens* because of the diversity of it (Pinto et al., 2007; Oliveira et al., 2015). Commonly, AHLs are synthesized by the bacterial LuxI-type enzymes and are bound to LuxR-type receptors to form receptor–AHL complexes, which associate with short DNA sequences termed “*lux boxes*” upstream of target genes to regulate diverse biological functions (Schuster et al., 2004). However, an increasing number of studies indicate that bacteria that do not produce, or produce fewer, QS signaling molecules can benefit from monitoring the density of environmental molecules, rather than investing effort into the production of “public goods” such as their own QS signaling molecules (Diggle et al., 2007). This phenomenon of using QS signaling molecules synthesized by other microorganisms to enhance their own collective behavior is known as “eavesdropping” in bacterial populations (Case et al., 2008). For example, Smith et al. (2011) demonstrated eavesdropping of AHLs by *Salmonella* promoted growth and survival in food environments. Similarly, Almeida et al. (2017) found that C_12_-HSL induced biofilm formation of *Salmonella enterica*, indicating these bacteria can respond to AHLs synthesized by other bacterial species. However, scarce information is available on this phenomenon in foodborne spoilage bacteria, including *P. fluorescens*, and its relationship with food spoilage.

Turbot (*Scophthalmus maximus*) is one of the most economically important aquaculture fish species in China. Turbot is vulnerable to microbial spoilage due to its soft texture and high moisture and protein content. Our previous study demonstrated that *P. fluorescens* is a SSO of turbot stored at 4°C and its spoilage potential has been demonstrated to be regulated by the QS system (Yang et al., 2017). Interestingly, AHL production of *P. fluorescens* used in this study is strikingly weaker, regardless of AHL type, than other microorganisms simultaneously isolated from spoiled turbot, such as *Aeromonas sobria* (Li et al., 2016). However, *P. fluorescens* is capable of becoming the SSO of turbot and demonstrates strong spoilage potential in later storage periods. Generally, microorganisms with strong QS tend to have better environmental stress tolerance and competitiveness due to up-regulated expression of related genes (Yuan et al., 2005; Bronesky et al., 2016). Therefore, the phenomenon of signaling molecule eavesdropping may also exist in *P. fluorescens* to improve its spoilage potential, especially in the condition that a variety of AHLs had been detected in spoiled turbots (Zhang et al., 2016a). Hence, the effects of various non-secreted AHLs on QS phenotypes (i.e., biofilm formation and ECP activity) and spoilage potential of *P. fluorescens* were evaluated in this study. Furthermore, changes in QS-related gene expression and the binding ability of exogenous AHLs with receptor proteins in *P. fluorescens* were analyzed.

## Materials and Methods

### Materials and Bacterial Strains

Live commercial-size (900 ± 5 g) turbots were purchased from a local Aquatic Market (Jinzhou, China) and transported to the Food Safety Key Lab of Liaoning Province for further experiments. All six AHL standards, *N*-butanoyl-L-homoserine lactone (C_4_-HSL), *N*-hexanoyl-L-homoserine lactone (C_6_-HSL), *N*-octanoyl-L-homoserine lactone (C_8_-HSL), *N*-decanoyl-L-homoserine lactone (C_10_-HSL), *N*-dodecanoyl-L-homoserine lactone (C_12_-HSL), and *N*-tetradecanoyl-L-homoserine lactone (C_14_-HSL) were purchased from Sigma-Aldrich (St. Louis, MO, United States), and dissolved in methanol as stock solutions with the concentration of 2 mg/mL.

In the subsequent experiments, the stock solution was diluted at 1:1000 ratio in the medium to the final concentration of 2 μg/mL (C_4_-HSL: 11.68 μM, C_6_-HSL: 10.04 μM, C_8_-HSL: 8.8 μM, C_10_-HSL: 7.83 μM, C_12_-HSL: 7.06 μM, C_14_-HSL: 6.42 μM). Other chemical agents were all of analytical grade and commercially available.

*Pseudomonas fluorescens* (GenBank accession number: CP032618) used in the present study was previously isolated from refrigerated turbots following the method of Yi et al. (2011) and was cultured overnight in Luria–Bertani (LB) broth (1% peptone, 1% NaCl, 0.5% yeast extract, pH 7.0 ± 0.2) at 160 rpm and 28°C, until an OD_600_ of 1 was reached.

### Assay of Bacterial Growth

The effect of different AHLs on the bacterial growth was evaluated according to the method of Jie et al. (2018). Overnight culture of *P. fluorescens* (OD_600_ = 1) was diluted at 1:100 ratio in fresh LB broth containing 2 μg/mL of different AHLs (C_4_-HSL, C_6_-HSL, C_8_-HSL, C_10_-HSL, C_12_-HSL, and C_14_-HSL), respectively. Equivalent methanol was added to *P. fluorescens* culture as the control group. Then, all samples were incubated at 28°C for 48 h. Bacterial density was estimated by measuring the OD_600_ in 3 h intervals.

### Assay of Biofilm Formation

Biofilm formation was assessed following the method described by Rode et al. (2007). First, 100 μL of *P. fluorescens* overnight culture was inoculated in the sterile LB broth containing 2 μg/mL of different AHLs (C_4_-HSL, C_6_-HSL, C_8_-HSL, C_10_-HSL, C_12_-HSL, and C_14_-HSL), respectively. Samples that were treated with equivalent methanol were used as control groups. Immediately, 1 mL culture containing AHLs was transferred to germ-free 1.5 mL microcentrifuge tube and further incubated statically at 28°C for 48 h. After discarding the suspension cultures, each microtube was rinsed thrice with sterile water to eliminate planktonic bacteria, and dried for 30 min. Then, 1 mL of 0.1% (w/v) crystal violet was used to stain the surface-adhered cells in the tubes for 15 min. Next, the tubes were washed thoroughly with sterile water and the crystal violet-stained biofilm was re-solubilized in 1 mL of 33% acetic acid, followed by transferring 200 μL of the above solution to a 96-well microtiter plate. Finally, the absorbance in each well at 595 nm was measured. Each sample was independently repeated nine times.

### Analysis of Biofilms by SEM

The SEM observation was performed as previously described (Gomes and Mergulhão, 2017), and *P. fluorescens* was cultured overnight in LB broth with shaking (160 rpm) at 28°C. Then, 100 μL of culture was diluted in 10 mL of sterile LB broth containing 2 μg/mL of different AHLs (C_4_-HSL, C_6_-HSL, C_8_-HSL, C_10_-HSL, C_12_-HSL, and C_14_-HSL). Next, a piece of polished zinc was placed in each dilution culture to facilitate biofilm formation. After incubation at 28°C for 48 h, the zinc pieces were removed from the cultures and rinsed thrice with sterile water. Immediately, the zinc pieces were immersed in 2.5% (v/v) precooled glutaraldehyde for another 4 h, and soaked in 50, 70, 80, and 90% (v/v) ethanol for 10 min, followed by twice incubation in 100% ethanol for 15 min. Finally, the biofilms were visualized by SEM, and samples without signaling molecules were used as control groups.

### Extracellular Protease Activity Assay

#### Milk Plate Assay

Extracellular protease activity in *P. fluorescens* was measured as previously described (Vijayaraghavan and Vincent, 2013). Briefly, 15% (w/v) skim milk and 1.5% (w/v) agar were prepared, sterilized at 115°C for 30 min, and cooled to 50°C. The milk agar plates were prepared by mixing skim milk and agar at a 1:1 ratio. Then, overnight culture of *P. fluorescens* (OD_600_ = 1) was diluted at 1:100 ratio in fresh LB broth containing 2 μg/mL of different AHLs (C_4_-HSL, C_6_-HSL, C_8_-HSL, C_10_-HSL, C_12_-HSL, and C_14_-HSL), respectively. After cultivation (28°C, 48 h), the culture was centrifuged at 8000 × *g* for 10 min. Immediately, the supernatant containing the extracellular protease was collected. Next, 200 μL of aliquot was added to the milk agar plates after filtering through a 0.22-μm filter and incubated for another 48 h. Finally, the size of the transparent enzymolysis zones was determined and samples without signaling molecules were used as control groups.

#### Azocasein Assay

Proteolytic activity of *P. fluorescens* culture was determined according to the method described by Martins et al. (2014) by using azocasein assay. Briefly, overnight cultures of *P. fluorescens* (OD_600_ = 1) were diluted at a 1:100 ratio in fresh LB containing 2 μg/mL of different AHLs. After culturing at 28°C for 48 h, different cultures were centrifuged at 8000 × *g* for 10 min. Thereafter, 150 μL of sterile filtered culture supernatant was added to 250 μL of 2% azocasein (w/v). After incubation (30°C for 12 h), this was mixed with 1.2 mL of 10% (w/v) trichloroacetic acid and the new mixture was further incubated at 25°C for 15 min. After centrifuging for 10 min at 15,000 × *g*, 600 μL of supernatant was removed and added to 50 μL of 1 M NaOH. Finally, the proteolytic activity of *P. fluorescens* culture was quantified by determining the OD_440_ of the mixture. Moreover, 150 μL of LB media was used as blank and the *P. fluorescens* culture without any AHLs was used as a control group. Each sample was used for three independent replicates.

### Detection of AHL Eavesdropping by GC–MS

#### AHL Extraction

*N*-Acyl homoserine lactones extraction was based on the procedure introduced by Ravn et al. (2001). First, 1 mL of *P. fluorescens* overnight culture (OD_600_ = 1) was inoculated in 100 mL of LB broth containing 2 μg/mL of different AHLs (C_4_-HSL, C_6_-HSL, C_8_-HSL, C_10_-HSL, C_12_-HSL, and C_14_-HSL), respectively, and cultured at 160 rpm and 28°C for only 24 h to avoid the alkaline environment and acylase accumulation caused by long-term culture. Moreover, as blank control, equal amounts (2 μg/mL) of different AHLs were added to sterile fresh LB broth (100 mL) and cultured under the same conditions. After incubation and centrifugation (8000 × *g*, 10 min), the supernatant was extracted thrice with 100 mL of ethyl acetate, followed by evaporating the organic phase in a rotary evaporator. Finally, the extract was dissolved in 1 mL of methanol and filtered through 0.22-μm filters before storage at −20°C for further analysis. AHLs in pure culture of *P. fluorescens* (without exogenous AHLs) were also extracted and detected by gas chromatography–mass spectrometry (GC–MS) to identify the composition of AHLs secreted by *P. fluorescens*.

#### GC–MS Conditions

The AHLs were further identified and quantified by GC–MS according to Zhu et al. (2016), with slight modification. GC–MS analyses were carried out on the GC–MS-7890N/5975 instrument (Agilent, Palo Alto, CA, United States) with a HP-5 MS capillary column (30 m length × 0.25 mm internal diameter × 0.25 μm film thickness). All sample injections were done in a splitless mode when the inlet temperature was maintained at 200°C, and pure helium (1.0 mL/min) was used as the carrier gas. When measuring AHLs, the GC oven temperature was programmed as follows: 150°C ramped at 10°C/min to 220°C, next ramped at 5°C/min to 250°C, and ramped at 0.5°C/min to 252.5°C. Mass spectra were obtained under the following conditions: electron ionization source was set to 70 eV, emission current 500 μA, MS Quad temperature 150°C, and MS Source temperature 230°C. Data were acquired by scanning the *m*/*z* range of 35–800 amu and by selected ion monitoring of *m*/*z* 143. AHLs in extracts were identified by comparing the retention times and chromatographic characteristics with each AHL standard.

### RNA Isolation, cDNA Synthesis, and Quantitative Real-Time PCR

Four QS-related genes (*rhlI*, *rhlR*, *flgA*, and *aprA*) were previously found in *P. fluorescens* by sequencing and whole genome sequence analysis of the test strain. Overnight culture of *P. fluorescens* (OD_600_ = 1) was diluted at 1:100 ratio in fresh LB broth containing 2 μg/mL of different AHLs (C_4_-HSL, C_6_-HSL, C_8_-HSL, C_10_-HSL, C_12_-HSL, and C_14_-HSL), respectively. Samples without signaling molecules were used as control groups. After cultivation (adjusted to 1.0 at OD_600_ nm), the total RNA of test strains was extracted according to the manufacturer’s instructions using TRIzol reagent (Sigma Chemicals), and treated twice with DNase I (Thermo Scientific, #EN0521). After using a Nanodrop to ensure an OD_260_/OD_280_ between 1.7 and 2.1, the RNA was reverse transcribed using RevertAid First Strand cDNA Synthesis Kit (Thermo Scientific, #K1621) following the manufacturer’s instructions. Finally, *Power* SYBR^®^ Green PCR Master Mix (Applied Biosystems^®^ Cat: 4367659) was used for performing quantitative real-time PCR (qPCR) analysis (CFX Connect^TM^ System, BIO-RAD, United States), under the following conditions: 95°C for 3 min, followed by 40 cycles of denaturation at 95°C for 10 s, annealing at 55°C for 20 s, and extension at 72°C for 20 s. The housekeeping gene 16S rRNA was used as an internal reference. The primer sequences are listed in Table 1. The expression level of different genes was quantified with the standard formula by calculating 2^–ΔΔCt^.

**TABLE 1:** Oligonucleotides used and PCR product sizes.

**Primer**	**PCR product size (bp)**	**Oligonucleotide sequence (5′–3′)**	**Use**
*rhlR* F1	155	GACCCCTTTTACCCGACCA	Objective gene
*rhlR* R1		CAAACAGTGCGTCGTTCCA	
*rhlI* F1	168	AAGTCTTCGGGTTTCTGTGC	Objective gene
*rhlI* R1		TCACTGCCACCACCTGATTA	
*flgA* F1	160	TGGAACAGGCAGAAGTGGT	Objective gene
*flgA* R1		CGAGCCTTGATCACACGTT	
*aprA* F1	84	CAACTCCAATGCCGACCGTGAG	Objective gene
*aprA* R1		CAACTCCAATGCCGACCGTGAG	
*16S rRNA F1*	133	GCTTTCGCCCATTGTCCAA	Reference
*16S rRNA R1*		TGGTGGGGTAATGGCTCAC	gene

*All primers for qPCR were designed with Primer 5.0 software and synthesized by Sagan (Shanghai, China).*

### *In silico* Analysis

Homology protein models of QS transcriptional activator (RhlR) were generated and assessed with SWISS-MODEL^1^ (Biasini et al., 2014; Waterhouse et al., 2018) according to the primary structure of RhlR protein. The 3D files of AHLs (C_4_-HSL, C_6_-HSL, C_8_-HSL, C_10_-HSL, C_12_-HSL, and C_14_-HSL) were obtained from the open chemistry database of NCBI^2^. Before docking the ligands into the active site, the 3D structure of the modeled protein was analyzed and further optimized by the addition of missing hydrogen atoms and removing excess crystallographic water using the SYBYL-X 2.1 program (Ding et al., 2017). Furthermore, spheres within 5 Å of the ligand were selected and defined as docking packets, and the residues not in the active site were deleted. Finally, the docking results were analyzed using the Cscore (Consensus Score) module of SYBYL-X 2.1 program to search for the best ligand–receptor conformations.

### Spoilage Potential of *P. fluorescens* in Turbot Filets

Turbot filets were prepared following the method of Dalgaard (1995), with slight modifications. First, the turbots were killed and fileted by a professional operator in “Lin Xi” aquatic market. Then, the filets were transport to the laboratory under aseptic conditions, followed by washing the filets with sterile water. After skinning the filets, the samples were wiped thoroughly with 75% (v/v) ethanol and exposed under an ultraviolet lamp until the total viable counts were <10^2^ CFU/g. For the preparation of inocula, cells of *P. fluorescens* cultured in LB broth (OD_600_ = 1) were collected by centrifugation at 8000 × *g* for 10 min, and the deposit was resuspended in sterile saline solution to dilute the bacterial concentration to 10^6^ CFU/mL. Finally, the turbot filets were immersed in the prepared bacterial suspension containing 2 μg/mL of different AHLs (C_4_-HSL, C_6_-HSL, C_8_-HSL, C_10_-HSL, C_12_-HSL, and C_14_-HSL), respectively, for 5 s to ensure the filets at a level of 10^4^ CFU/g. All filets were individually packed and divided into five groups (three subsamples in each group) stored at 4 ± 1°C. The total volatile basic nitrogen (TVB-N) (mg N/100 g muscle) contents were evaluated at 3-day intervals (0, 3, 6, 9, and 12 days, corresponding to five groups) to analyze the spoilage potential of *P. fluorescens.* Furthermore, the TVB-N changes of turbots without inoculating *P. fluorescens* were also determined as blank control.

For TVB-N analysis, each subsample was ground individually using a handheld blender (WBL25B26, Midea, China). Then, 10 g of ground filets was taken from each subsample, respectively, and added to a distilling tube containing 1 g of MgO and 50 mL of distilled water. The TVB-N contents of each subsample were determined using a Kjeltec 8400 automatic nitrogen determination apparatus (FOSS, Hiller, Denmark) in the Kjeldahl mode. The experimental results of each group were the average of three subsamples.

### Statistical Analysis

Each test was performed for three independently repeated experiments and data were presented as the mean ± standard deviation (SD). Statistical analysis was conducted by repeated measures using SPSS 20.0, and differences in mean values were tested for significance using one-way analysis of variance (ANOVA) with Tukey’s test. The level of statistical significance was determined at *P* < 0.05.

## Results

### The Effect of Exogenous AHLs on the Growth of *P. fluorescens*

The effects of six synthesized AHLs on the growth of *P. fluorescens* in LB broth were evaluated (Supplementary Figure S1). The results showed that the growth of *P. fluorescens* was significantly (*P* < 0.05) accelerated by shortening the lag phase in the presence of C_4_-HSL, C_6_-HSL, C_8_-HSL, C_12_-HSL, and C_14_-HSL. Moreover, the addition of most exogenous AHLs increased the maximum cell density of *P. fluorescens* during the 48 h, which demonstrated the promotion effect of AHLs on the growth of *P. fluorescens.* The results also showed that the treatment of C_10_-HSL had no significant effect on the bacterial growth.

### The Effect of Exogenous AHLs on Biofilm Formation in *P. fluorescens*

The effects of exogenous AHLs on biofilm formation in *P. fluorescens* were investigated by crystal violet assay (Supplementary Table S1). Addition of exogenous AHL molecules significantly enhanced biofilm formation of *P. fluorescens* (*P* < 0.05), except for C_10_-HSL. A maximum biofilm stimulation rate of 127.49% was obtained with C_4_-HSL, suggesting that exogenous AHLs can promote biofilm formation in *P. fluorescens*.

**FIGURE 1:**
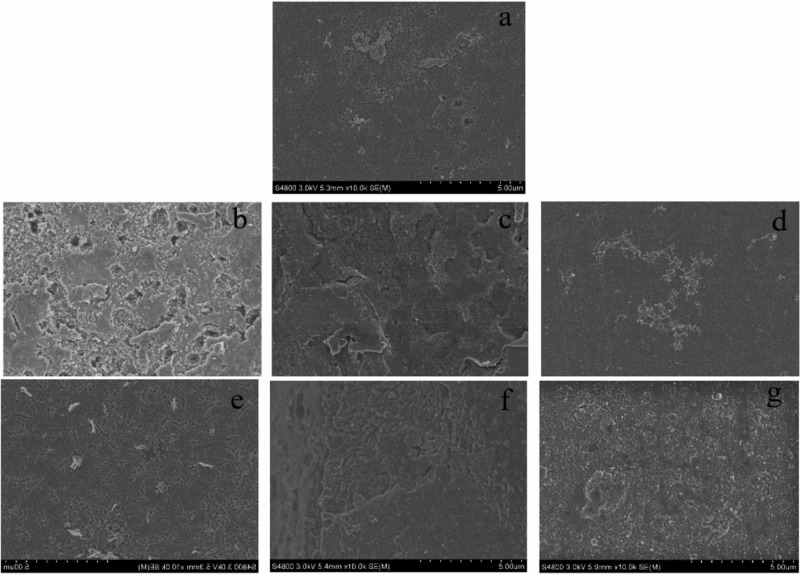
SEM images of biofilm formation in *P. fluorescens* after incubation with or without exogenous AHLs for 48 h. **(a)** Control and **(b–g)** AHLs treatment with C_4_-HSL, C_6_-HSL, C_8_-HSL, C_10_-HSL, C_12_-HSL, and C_14_-HSL at 2 μg/mL.

The images obtained by SEM further support these findings (Figure 1). The biofilm of samples treated with C_4_-HSL, C_6_-HSL, C_12_-HSL, and C_14_-HSL were thicker and more layered than that of the control group, confirming the promotion of biofilm formation in *P. fluorescens* by utilizing exogenous AHLs. Moreover, no significant effect on biofilm formation in *P. fluorescens* was observed in the presence of C_10_-HSL, consistent with the crystal violet assay.

### The Effect of Exogenous AHLs on the Protease Activity of *P. fluorescens*

**FIGURE 2:**
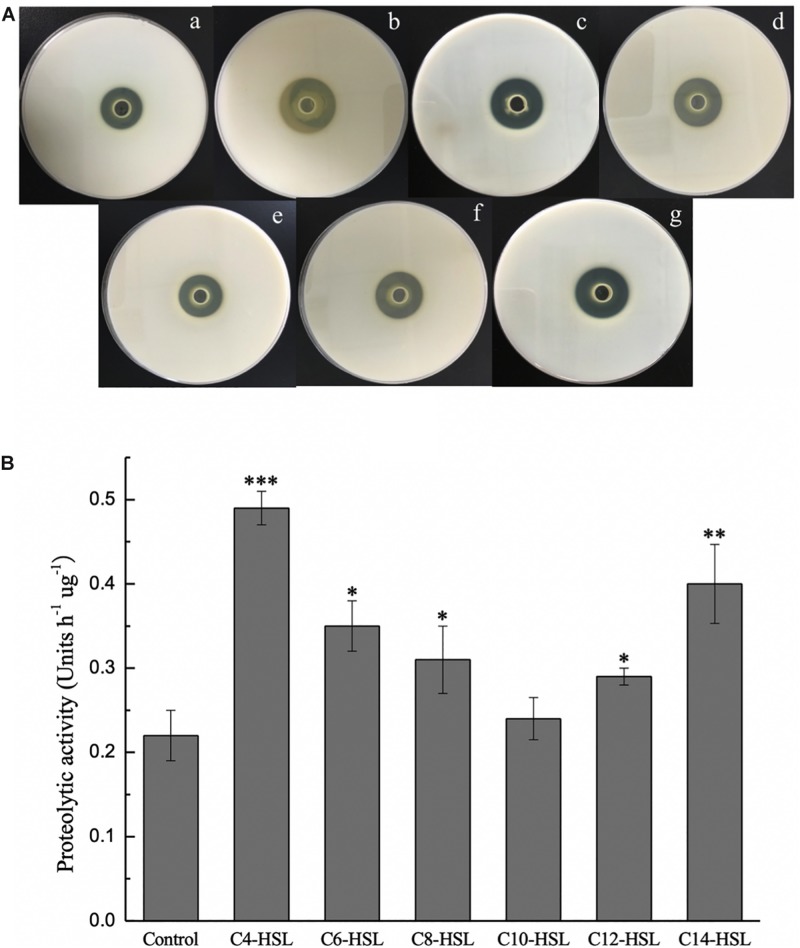
Extracellular proteinase activity of *P. fluorescens* after incubation with or without exogenous AHLs for 48 h tested by milk agar plates assay **(A)** and azocasein assay **(B)**. **(a)** Control and **(b–g)** AHLs treatment with C_4_-HSL, C_6_-HSL, C_8_-HSL, C_10_-HSL, C_12_-HSL, and C_14_-HSL at 2 μg/mL. Data are presented as means ± SD (*n* = 3, ^∗^*P* < 0.05, ^∗∗^*P* < 0.01, ^∗∗∗^*P* < 0.001).

As shown in Figure 2A, the effect of exogenous AHLs on extracellular protease secretion by *P. fluorescens* was studied using milk agar plates. A larger enzymolysis circle was clearly observed in the presence of C_4_-HSL, C_6_-HSL, C_8_-HSL, C_12_-HSL, and C_14_-HSL, confirming the effective utilization of AHLs by *P. fluorescens*. Treatment with C_10_-HSL had no significant effect (*P* > 0.05). Among all the exogenous AHLs, C_4_-HSL and C_14_-HSL had more significant stimulation rates at 63.40 and 52.42%, respectively (Supplementary Table S2). Similar to the results of milk plate assay, the proteolytic activity in *P. fluorescens* was also promoted by the addition of AHLs, indicating that this phenotype can be regulated by exogenous AHLs in the test strain (Figure 2B).

### Detection of AHL Eavesdropping by GC–MS

GC–MS analysis was performed to identify the AHLs secreted by *P. fluorescens*, by comparing the chromatographic characteristics of different synthetic AHL standards. As shown in Supplementary Figure S2, C_4_-HSL and C_10_-HSL were verified from the supernatant of *P. fluorescens*, with C_4_-HSL was the predominant AHL type. Meanwhile, the AHLs contents in sterile LB broth and *P. fluorescens* culture were also determined by GC–MS after incubation in the same condition. As shown in Table 2, the AHLs contents in sterile LB broth did not change significantly before and after the cultivation, which indicated that exogenous AHLs could be stable in the process of cultivation without apparent degradation. In contrast, a reduction in AHL contents was observed in *P. fluorescens* cultures, suggesting that *P. fluorescens* could enhance the QS behavior by eavesdropping exogenous AHLs. Furthermore, the utilization of C_14_-HSL by *P. fluorescens* was the most significant, followed by C_8_-HSL and C_6_-HSL (Table 2).

**TABLE 2:** The concentration changes of exogenous AHLs in different cultures during incubation (mean ± SD).

**Additive**	**Concentration before culture^a^ (μg/mL)**	**Concentration after culture (μg/mL)^b^**		**Consumption rate (%)^c^**	
			
		**Sterile broth**	**Bacterial culture**	**Sterile broth**	**Bacterial culture**
C_4_-HSL	2^A^	1.934 ± 0.052^A^	1.351 ± 0.071^B^	3.30	32.45
C_6_-HSL	2^A^	1.891 ± 0.037^A^	0.925 ± 0.052^B^	5.45	53.75
C_8_-HSL	2^A^	1.903 ± 0.035^A^	0.747 ± 0.131^B^	4.85	62.65
C_10_-HSL	2^A^	1.874 ± 0.091^AB^	1.731 ± 0.035^B^	6.30	13.45
C_12_-HSL	2^A^	1.875 ± 0.067^A^	1.003 ± 0.095^B^	6.25	49.85
C_14_-HSL	2^A^	1.871 ± 0.082^A^	0.350 ± 0.073^B^	6.45	82.50

*^*a*^Expressed as the original addition amount of AHLs in sterile broth or *P. fluorescens* cultures before incubation at 28°C for 24 h. ^*b*^Expressed as the remaining amount of AHLs in sterile broth or *P. fluorescens* cultures after incubation at 28°C for 24 h. ^*c*^Consumption rate = [(concentration before culture − concentration after culture)/concentration before culture] × 100. ^*A,B*^In the same row with different superscripts are significantly different (*P* < 0.05).*

### The Effect of Exogenous AHLs on QS-Related Gene Expression of *P. fluorescens*

**FIGURE 3:**
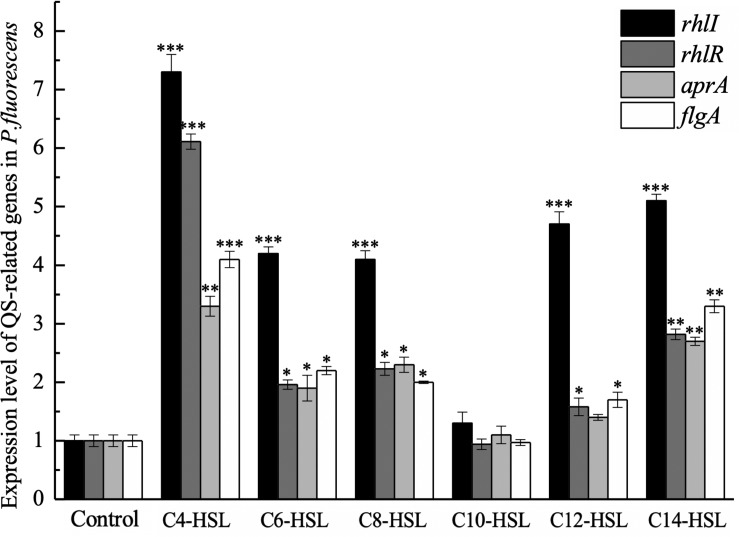
Expression level of QS-related genes in *P. fluorescens* after incubation with 2 μg/mL exogenous AHLs until an optical density at 600 nm (OD_600_) of 1 was reached. Data are presented as means ± SD (*n* = 3, ^∗^*P* < 0.05, ^∗∗^*P* < 0.01, ^∗∗∗^*P* < 0.001).

The relative expression of *rhlR* was examined to explore whether *P. fluorescens* could sense AHLs in the environment. The result of qPCR analysis suggests that the AHLs used in this study can upregulate the expression level of *rhlR* in *P. fluorescens*, except for C_10_-HSL (Figure 3). As shown in Figure 3, C_4_-HSL and C_14_-HSL were the most effective AHLs in upregulating *rhlR* expression, 6.11-fold and 2.18-fold, respectively. Furthermore, the expression of *rhlI*, *flgA*, and *aprA* was also analyzed. Consistent with the results of the *rhlR*, the addition of most AHLs stimulated expression of these genes, but C_10_-HSL had no significant effect.

### Homology Modeling and Model Quality Estimation

The RhlI/R system is known as one of the typical QS systems in *Pseudomonas* sp. and was the only QS system annotated in the genome of the test strain (GenBank accession number: CP032618). Meanwhile, RhlR protein, as the specific receptor of C_4_-HSL in this system, can regulate several biological functions of *Pseudomonas* sp. (Yan et al., 2007). In this study, 3D models of RhlR protein in *P. fluorescens* were generated using SWISS-MODEL in the first approach mode, and the obtained models were estimated by using the Protein Structure & Model Assessment Tools in SWISS-MODEL. Sequence alignment results showed that the two models of 4y13.1.A and 3qp5.1.A had high similarity (>35%) with the RhlR protein sequence of *P. fluorescens* in all 50 template sequences matched by the Template Library of SWISS-MODEL, based on sequence similarities and the Global Model Quality Estimation (GMQE) score (Supplementary Figure S3).

The primary structure of a protein is the basis of its 3D structure, and so, two sequences with homologous relationships usually have similar spatial structures. Therefore, homology models generated based on the two template sequences were shown in Supplementary Table S3 and Qualitative Model Energy Analysis (QMEAN), a comprehensive scoring function for model quality assessment was used to assess the entire model (Benkert and Tosatto, 2008). The nearest 0 score conforms a high quality for the model, whereas −4.0 or lower scores are indicated as low quality. The result showed that the model of 4y13.1.A was selected as the optimal model of RhlR for further analysis, due to the higher GMQE score (0.75) and QMEAN score (−2.31) (Supplementary Table S3). Furthermore, 4y13.1.A was also closer to the central dot, representing the target protein RhlR, in the visual map of the model comprehensive evaluation, which indicates that model 4y13.1.A can predict the 3D structure of RhlR protein well (Supplementary Figure S4).

### Docking Analysis

**FIGURE 4:**
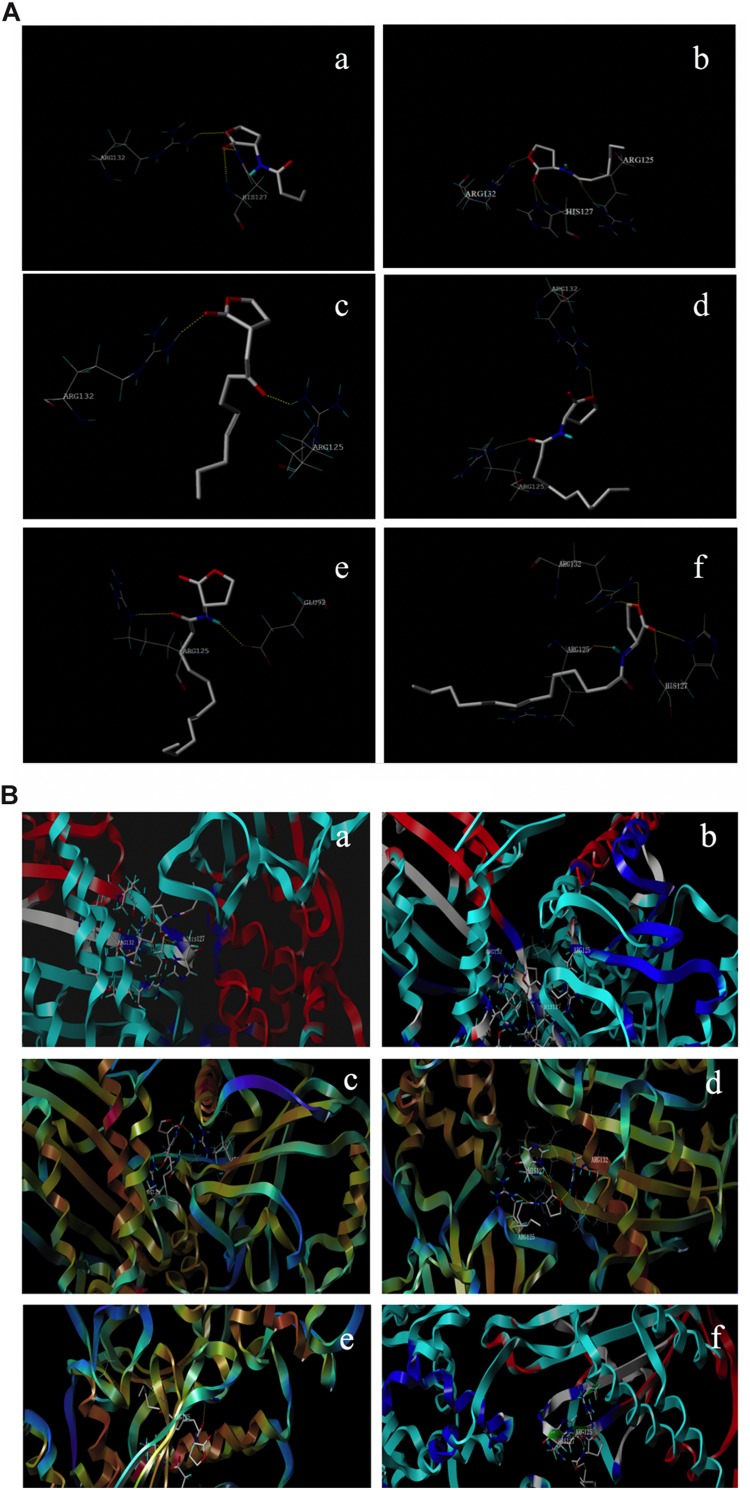
Interaction of different AHLs with the active site of RhlR type proteins in *P. fluorescens* **(A)** and the predicted conformation of AHLs in the binding packet **(B)**. **(a–f)** C_4_-HSL, C_6_-HSL, C_8_-HSL, C_10_-HSL, C_12_-HSL, and C_14_-HSL.

The interaction of AHLs–RhlR was analyzed using computer-aided molecular docking. The protein model selected above (4y13.1.A) was prepared and docked with AHL molecules using the SYBYL-X 2.1 program. As shown in Figure 4A, all different AHL molecules bound to the active sites of the RhlR-type protein by hydrogen bonding, indicating the binding potential of signaling molecules to the RhlR protein in *P. fluorescens*. Furthermore, the Cscores of all six ligands were not less than 4, which realistically reflected the space conformation of the AHL–RhlR complex (Supplementary Table S4).

### Effects of Exogenous AHLs on the Spoilage Process of Refrigerated Turbot Filets

**FIGURE 5:**
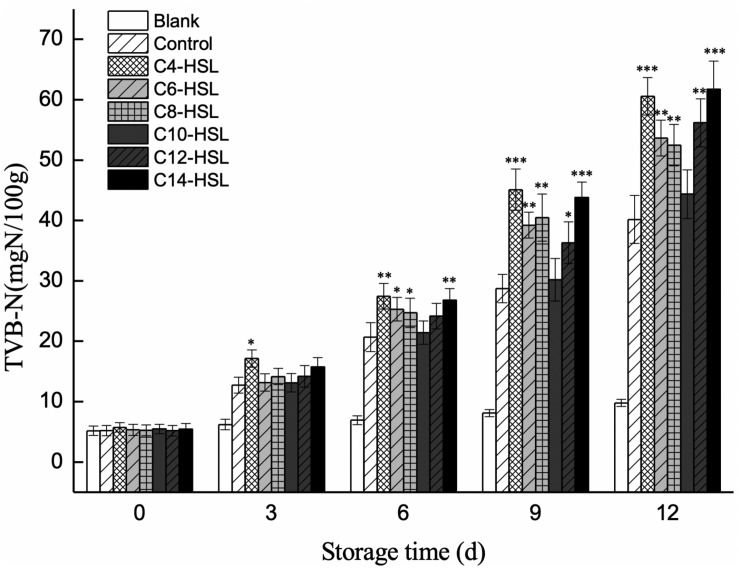
Changes in the TVB-N value of turbot filets contaminated with different AHLs during refrigerated storage. Data are presented as means ± SD (*n* = 3, ^∗^*P* < 0.05, ^∗∗^*P* < 0.01, ^∗∗∗^*P* < 0.001).

The production of TVB-N by *P. fluorescens* in turbot filets was monitored to evaluate the potential involvement of exogenous AHLs in the spoilage process. The results showed an increase in TVB-N values during storage, where the increased levels were significantly influenced by the presence of exogenous AHLs (Figure 5). TVB-N value increased from 5.21 to 40.17 mg N/100 g in the control group. Comparing all the AHLs added, exogenous C_4_-HSL and C_14_-HSL exhibited the greatest impact on the promotion of TVB-N values at the end of storage, reaching final values of 60.57 and 61.73 mg N/100 g, respectively.

## Discussion

The QS system, mediated by AHL signaling molecules, is prevalent among diverse bacterial species, particularly in gram-negative bacteria. The RhlI/R system is a common AHL-mediated QS system discovered in *P. aeruginosa*. As a transcriptional regulator, RhlR can specificity bind C_4_-HSL (synthesized by RhlI) to promote the expression of downstream genes, regulating biofilm formation, rhamnolipid biosynthesis, and other behaviors (Maier and Soberón-Chávez, 2000). However, the RhlI/R system in *P. fluorescens* and its regulatory effect are rarely reported. Furthermore, the QS system of microorganisms based on interactions between exogenous signaling molecules and receptor proteins has attracted attention (Cheng et al., 2018; Lv et al., 2018). Therefore, further research is still needed to evaluate the involvement of exogenous signaling molecules in the spoilage potential of bacteria, though the effects of the AHL-related QS system on aquatic product spoilage have been confirmed (Fu et al., 2018). The present study aimed to explore the effect of exogenous signaling molecules on QS-mediated spoilage of turbot by *P. fluorescens* via the *rhl*-system.

It has been reported that the QS system may play a positive role in the growth of some bacteria, such as *Shewanella putrefaciens*, *Shewanella baltica*, and *P. aeruginosa*, in order to improve their competitiveness in response to the environmental change (Zhang et al., 2014; Zhu et al., 2015). This may be due to the beneficial effects of some metabolites regulated by QS on cell division. Interestingly, it has been shown that some QS-deficient mutant bacteria which lack QS-controlled factors display growth advantages relative to wild strains under certain conditions (Sandoz et al., 2007). Likely, this phenomenon is due to “cheaters,” which can directly utilize, rather than produce, “public goods” such as AHLs. This reduces the metabolic burden for these species under conditions that ensure normal metabolism. Because of the importance of QS on microbial growth, different exogenous AHLs were added to the cultures of *P. fluorescens* to analyze their effect on bacterial growth. As shown in Supplementary Figure S1, exogenous AHLs successfully elevated population density levels of *P. fluorescens* and accelerated bacterial growth. This effect has been previously reported by many researchers (Nychas et al., 2009; Dourou et al., 2011). Our previous study also found that exogenous C_4_-HSL and C_8_-HSL accelerated growth and increased cell density of *A. sobria* AS7 in turbot (Li et al., 2016). The results obtained here confirmed a similar effect of AHLs on *P. fluorescens*. As previously discussed, this may be because exogenous AHLs stimulated the QS system in *P. fluorescens*, thus enhancing the production of growth-beneficial factors. It may also be that exogenous AHLs reduced the metabolic burden on *P. fluorescens*. Notably, some researchers also believe that phenomenon is observed because AHLs are used by bacteria as a carbon source. While it is difficult to fully define the causes of this phenomenon, the results obtained here suggested that *P. fluorescens* have the ability of utilizing non-secreted AHLs to regulate their growth. These findings agreed with those of Zhu et al. (2018), who showed that exogenous AHLs can be utilized by *S. baltica* through the LuxR receptor to promote growth.

The selective pressure of antimicrobials or preservatives forces microorganisms to evolve a series of resistance mechanisms to ensure the species survival; among these, biofilm formation is one of the most effective strategies and is regulated by the QS system. Furthermore, in the food industry, microbial biofilms usually pose potential safety risks to food processing due to their adhesiveness ability to the contact surfaces of food processing equipment. Therefore, the ability of biofilm formation of spoilage bacteria can lead to the potential risk of biofilm-food cross-contamination in food processing. Previously, Davies et al. (1998) demonstrated the necessity of self-secreted signaling molecules in the development of *P. aeruginosa* biofilms by constructing *lasI* or *rhlI* mutants. In this study, the positive regulation of non-secreted AHLs on biofilm formation in *P. fluorescens* was further verified via the crystal violet assay (Supplementary Table S1), which showed that the addition of C_4_-HSL, C_6_-HSL, C_8_-HSL, C_12_-HSL, and C_14_-HSL promoted biofilm production in *P. fluorescens*. Moreover, the thicker biofilm images (Figures 1b,c,g) were also observed by SEM, indicating the treatment of exogenous AHLs stimulated the production of extracellular matrix in *P. fluorescens*. GC–MS results showed that the *P. fluorescens* used in this study could not produce C_6_-HSL and C_14_-HSL (Supplementary Figure S2), which confirmed the ability of *P. fluorescens* to promote biofilm formation by using exogenous signaling molecules in the environment. Similarly, Almeida et al. (2017) also confirmed the obvious effect of exogenous AHLs on biofilm production via the LuxR homolog regulator – SdiA. This is also consistent with the findings of Kusada et al. (2014), who found that the biofilm formation in *Acidovorax* sp. strain MR-S7 was induced by different kinds of AHL, in which 3-oxo-C_8_-HSL and C_10_-HSL had a better promotion rate.

As one of the major QS-mediated metabolites of bacteria, extracellular protease is considered to be largely responsible for the spoilage of aquatic products. Accordingly, the protease activity of *P. fluorescens* after treatment with various AHLs was evaluated to further explain the relationship between the eavesdropping phenomenon and the spoilage potential of bacteria. As shown in Figure 2A, *P. fluorescens* secreted extracellular proteases and produced clear protein lysis circles in milk agar plates during culture. Upon addition of exogenous signaling molecules, the protease activity of *P. fluorescens* increased relative to the control group. The proteolytic activity in *P. fluorescens* was also determined by an azocasein assay, with the unit of enzyme activity per hour per gram of protein defined as one unit of proteolytic activity. The results demonstrated that proteolytic activity in *P. fluorescens* treated with C_4_-HSL and C_1__4_-HSL increased significantly (*P* < 0.05) compared to the control group, with the proteolytic activity reaching 0.42 and 0.40 h^–1^μg^–1^, respectively. Moreover, the proteolytic activity of the other samples also increased, except for C_10_-HSL group (Figure 2B). These results are in agreement with the observations of Zhu et al. (2015), who found that C_14_-HSL, 3-oxo-C_6_-HSL, 3-oxo-C_8_-HSL, and cyclo-(L-Pro-L-Leu) increased the extracellular proteolytic activity of *S. baltica* SA02, whereas a contrary effect was found with the addition of C_6_-HSL, C_8_-HSL, C_10_-HSL, and C_12_-HSL. Interestingly, Martins et al. (2014) showed that AHLs have no effect on the proteolytic activity in *P. fluorescens* 07A and 041, and even these two strains do not produce AHLs. These authors attributed this to the diversity of *P. fluorescens*, which the results of this paper undoubtedly confirmed this again.

As previously mentioned, AHLs secreted by bacteria can specifically bind to receptors, resulting in expression of downstream target genes and changes to their own composition. Accordingly, GC–MS was used to monitor the content changes of added exogenous AHLs in *P. fluorescens* cultures to determine whether *P. fluorescens* was directly capable of AHLs eavesdropping. Culture time was strictly controlled at 24 h to avoid the negative effects of the alkaline environment and the accumulation of acylase on the AHLs that occur with extended culturing (>30 h) (data not shown). GC–MS verified the eavesdropping of *P. fluorescens* on exogenous AHLs, with an 82.5% reduction of C_14_-HSL after culturing with *P. fluorescens* for 24 h (Table 2). Cultures without *P. fluorescens* showed no significant AHLs depletion. Similarly, the determination of β-galactosidase by Zhu et al. (2018) revealed that *S. baltica* could eavesdrop on the ambient AHLs produced by *Acinetobacter*, and AHL activity of pure *Acinetobacter* culture was significantly higher than that of the co-culture of *S. baltica* and *Acinetobacter* with an inoculation ratio of 1:1. Notably, the content of C_4_-HSL in *P. fluorescens* cultures did not change dramatically after incubation compared to that of C_14_-HSL, although C_4_-HSL plays the most important role in biofilm and protease experiments. This finding is likely due to the continuous supplementation of C_4_-HSL into the media via production by *P. fluorescens* (Supplementary Figure S2). Moreover, the content of C_10_-HSL in *P. fluorescens* did not change significantly before and after incubation, with the concentration maintained at 1.731 μg/mL, which was the highest among the six AHLs. This suggested that C_10_-HSL was not used by *P. fluorescens*, which was consistent with the results of the biofilm and protease assays.

Previously, the whole genome of the test strain was sequenced and analyzed by Novogene Bioinformatics Technology Co., Ltd. (Beijing, China) to identify the QS-related genes in the *P. fluorescens* used in this study. The gene functions were predicted (*E*-value <1*e*−5, minimal alignment length percentage >40%) based on Gene Ontology (GO), Non-redundant Protein Database (NR), and Kyoto Encyclopedia of Genes and Genomes (KEGG) databases. Further, the predictive function of selected genes was verified by searching in the GenBank sequence database^3^. Finally, the AHLs synthetase gene (*rhlI*), QS signaling molecules receptor protein gene (*rhlR*), spoilage-related alkaline proteinase gene (*aprA*), and biofilm-related flagellar gene (*flgA*) were found in *P. fluorescens*.

In this study, the effect of synthesized AHLs on the transcription of *rhlI*, *rhlR*, *aprA*, and *flgA* was further measured using qPCR to evaluate whether the promotion of biofilm formation and protease activity by exogenous AHLs was related to the *rhl*-mediated QS system in *P. fluorescens*. The results verified that C_4_-HSL, C_6_-HSL, C_8_-HSL, C_12_-HSL, and C_1__4_-HSL promoted the transcription level of these genes, which indicated that *P. fluorescens* could sense the exogenous AHLs in the environment. Further, upregulation of QS genes caused by exogenous AHLs could explain the promotion of biofilm formation and protease activity in *P. fluorescens*. However, the *rhlR* transcription was slightly downregulated in response to C_10_-HSL, suggesting a specific selectivity of AHLs in regulating QS system in *P. fluorescens*. Similarly, Zhu et al. (2016) indicated that cyclo-(L-Pro-L-Leu) as exogenous autoinducers promoted the transcription levels of *luxR* and *torA* in both *S. baltica* 02 and *S. baltica* 08, while no significant changes were observed in the presence of cyclo-(L-Pro-L-Phe) or 4,5-dihydroxy-2,3-pentanedione.

The results of qPCR in this study verified the expression change of *rhlR* caused by exogenous AHLs. Therefore, the computer-aided molecular docking technique which has been widely applied to the prediction of binding-conformation of small molecule ligands to their receptors was used to explore the recognition and interaction of RhlR-type protein with AHLs in *P. fluorescens*. The docking results confirmed that all exogenous AHLs, like the native ligand C_4_-HSL, rigidly docked into the RhlR binding pocket (Figure 4B). The results suggested that the RhlR protein in *P. fluorescens* could combine with environmental signaling molecules. Interestingly, the docking scores of C_4_-HSL and C_14_-HSL were lower than those of others. This indicated that the binding ability of C_4_-HSL and C_14_-HSL to RhlR protein is not better than other AHLs, whereas C_10_-HSL exhibited the strongest binding affinity with RhlR protein (Supplementary Table S4).

Levels of TVB-N mainly reflect the accumulation of biogenic amines and other compounds derived from protein degradation by bacteria and are thus regarded as an essential quality parameter for assessing aquatic products spoilage, especially during cold storage. In this study, the regulation of exogenous AHLs on spoilage potential was measured by monitoring the production of TVB-N by *P. fluorescens* in turbot filets. As shown in Figure 5, the initial TVB-N values were similar in all the samples. However, the production of TVB-N was significantly stimulated in the presence of exogenous C_4_-HSL, C_6_-HSL, C_8_-HSL, C_12_-HSL, and C_14_-HSL, while no significant effect of exogenous C_10_-HSL was observed. On day 9, the TVB-N value of C_4_-HSL treated groups reached 45.11 mg N/100 g, which exceeded the acceptable limit of 30 mg N/100 g for marine fish products (Ding et al., 2014). Furthermore, the results also showed that the TVB-N value of blank control always kept at a low level, indicating that other bacteria contributed less to the spoilage of turbots. The result indicated the crucial role of environmental AHLs in the spoilage process of turbot caused by *P. fluorescens.* These results also fall in line with those of Zhang et al. (2016b), who found that C_4_-HSL and C_6_-HSL significantly increased the production of TVB-N in vacuum-packaged farmed turbot, while the addition of exogenous 3-oxo-C_6_-HSL had no significant effect. Similarly, Zhao et al. (2017) indicated that the addition of exogenous AHLs promoted the production of TVB-N by *Aeromonas veronii* in surimi juice.

## Conclusion

In conclusion, this study validated the involvement of environmental AHLs on turbot spoilage via the promotion of QS phenotypes in *P. fluorescens*, which are required for fish spoilage. Furthermore, an interesting potential utilization ability of environmental AHLs by *P. fluorescens* was found based on the results of qPCR and molecular docking analysis. Although this utilization should be further elucidated by constructing mutant strains deficient in the RhlR receptor, the results obtained here still provide insight into the role of AHLs eavesdropping in QS-based spoilage regulation of *P. fluorescens.* Moreover, the study also suggested that some QS inhibitors, such as some AHLs-quenching enzymes, should not focus solely on the degradation of AHLs secreted by SSO and the results could help formulate new strategies for QS control to further reduce the post-harvest loss of aquatic products.

## Data Availability Statement

The datasets generated for this study can be found in the GenBank accession number: CP032618.

## Author Contributions

TL conceived and designed the experiments, analyzed the data, contributed reagents, materials, and analysis tools, authored or reviewed drafts of the manuscript, and approved the final draft. DW and LR conceived and designed the experiments, performed the experiments, analyzed the data, prepared the figures, and/or tables, and authored or reviewed drafts of the manuscript. HC performed the GC–MS analysis. YM prepared the figures and/or tables. TD performed the docking molecular docking analysis. QL authored or reviewed drafts of the manuscript. JL conceived and designed the experiments, approved the final draft, and had overall responsibility for this project.

## Conflict of Interest

The authors declare that the research was conducted in the absence of any commercial or financial relationships that could be construed as a potential conflict of interest.

## Footnotes

1 https://swissmodel.expasy.org

2 https://pubchem.ncbi.nlm.nih.gov/

3 http://www.ncbi.nlm.nih.gov/
