# Supplementary material for: Involvement of Exogenous N-Acyl-Homoserine Lactones in Spoilage Potential of Pseudomonas fluorescens Isolated From Refrigerated Turbot
Source: Front Microbiol. 2019 Nov 29;10:2716. doi: 10.3389/fmicb.2019.02716 (PMC6895499; doi:10.3389/fmicb.2019.02716)

Table S1. The effects of exogenous AHL molecules on biofilm formation in *P. fluorescens* (mean ± SD).

| Additive | Concentration | Biofilm formation^a^ | Stimulation rate (%)^b^ |
| --- | --- | --- | --- |
| Control | 0 μL/mL | 0.793±0.021^g^ | — |
| C_4_-HSL | 2 μg/mL | 1.804±0.013^c^ | 127.49% |
| C_6_-HSL | 2 μg/mL | 1.212±0.025^e^ | 52.83% |
| C_8_-HSL | 2 μg/mL | 1.182±0.009^e^ | 49.05% |
| C_10_-HSL | 2 μg/mL | 0.814±0.021^g^ | 2.64% |
| C_12_-HSL | 2 μg/mL | 0.915±0.010^f^ | 15.38% |
| C_14_-HSL | 2 μg/mL | 1.481±0.031^d^ | 86.76% |

^a^Expressed as OD_595_ after staining with crystal violet

^b^The stimulation rate = [(OD experimental group – OD control group) / OD control group] ×100

^c-g^Significantly different means (*P* < 0.05)

Table S2. The effect of AHLs on protease activity of *P. fluorescens* (mean ± SD)

| Additive | Concentration | Proteinase activity (mm)^a^ | | Stimulation rate (%)^b^ |
| --- | --- | --- | --- | --- |
| Control | 0 μL/mL | 20.03±0.05^g^ | — | |
| C_4_-HSL | 2 μg/mL | 32.73±0.09^c^ | 63.40% | |
| C_6_-HSL | 2 μg/mL | 27.31±0.07^e^ | 36.35% | |
| C_8_-HSL | 2 μg/mL | 26.17±0.11^e^ | 30.65% | |
| C_10_-HSL | 2 μg/mL | 21.31±0.17^g^ | 6.39% | |
| C_12_-HSL | 2 μg/mL | 23.45±0.05^f^ | 17.07% | |
| C_14_-HSL | 2 μg/mL | 30.53±0.19^d^ | 52.42% | |

^a^Expressed as diameter of the transparent enzymolysis circle generated by *P. fluorescens* in milk agar plates.

^b^The stimulation rate = [(OD experimental group – OD control group) / OD control group] ×100

^c-g^Significantly different means (*P* < 0.05)

Table S3. Structures and scores of *P. fluorescens* RhlR proteins models

| Proteins | Template | Description | GMQE | QMEAN | Model |
| --- | --- | --- | --- | --- | --- |
| RhlR-type | 4y13.1.A | Transcriptional regulator of ftsQAZ gene cluster | 0.75 | -2.31 | 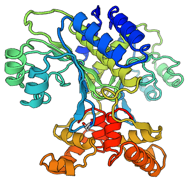 |
|  | 3qp5.1.A | CviR transcriptional regulator | 0.45 | -4.15 | 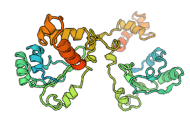 |

Table S4. Docking results of RhlR type protein of *P. fluorescens* with AHLs

| Protein | Ligand | Total score | Crash^a^ | Polar^b^ | Cscore^c^ |
| --- | --- | --- | --- | --- | --- |
| RhlR type | C_4_-HSL | 4.09 | -0.85 | 2.83 | 4 |
|  | C_6_-HSL | 7.87 | -0.69 | 3.09 | 4 |
|  | C_8_-HSL | 7.11 | -1.11 | 1.69 | 4 |
|  | C_10_-HSL | 8.67 | -2.06 | 3.08 | 4 |
|  | C_12_-HSL | 8.16 | -1.55 | 2.06 | 4 |
|  | C_14_-HSL | 6.94 | -2.89 | 3.00 | 4 |

^a^Crash represents the degree of inappropriate penetration by the ligand into the protein

^b^Polar represents the contribution of hydrogen bonding and salt bridge interactions to the total score

^c^ Cscore is the function for ranking the binding affinity of ligands to the active site of a receptor

Fig. S1. The effect of exogenous AHL molecules on the growth of *P. fluorescens* at 28°C. Data are presented as means ± SD.


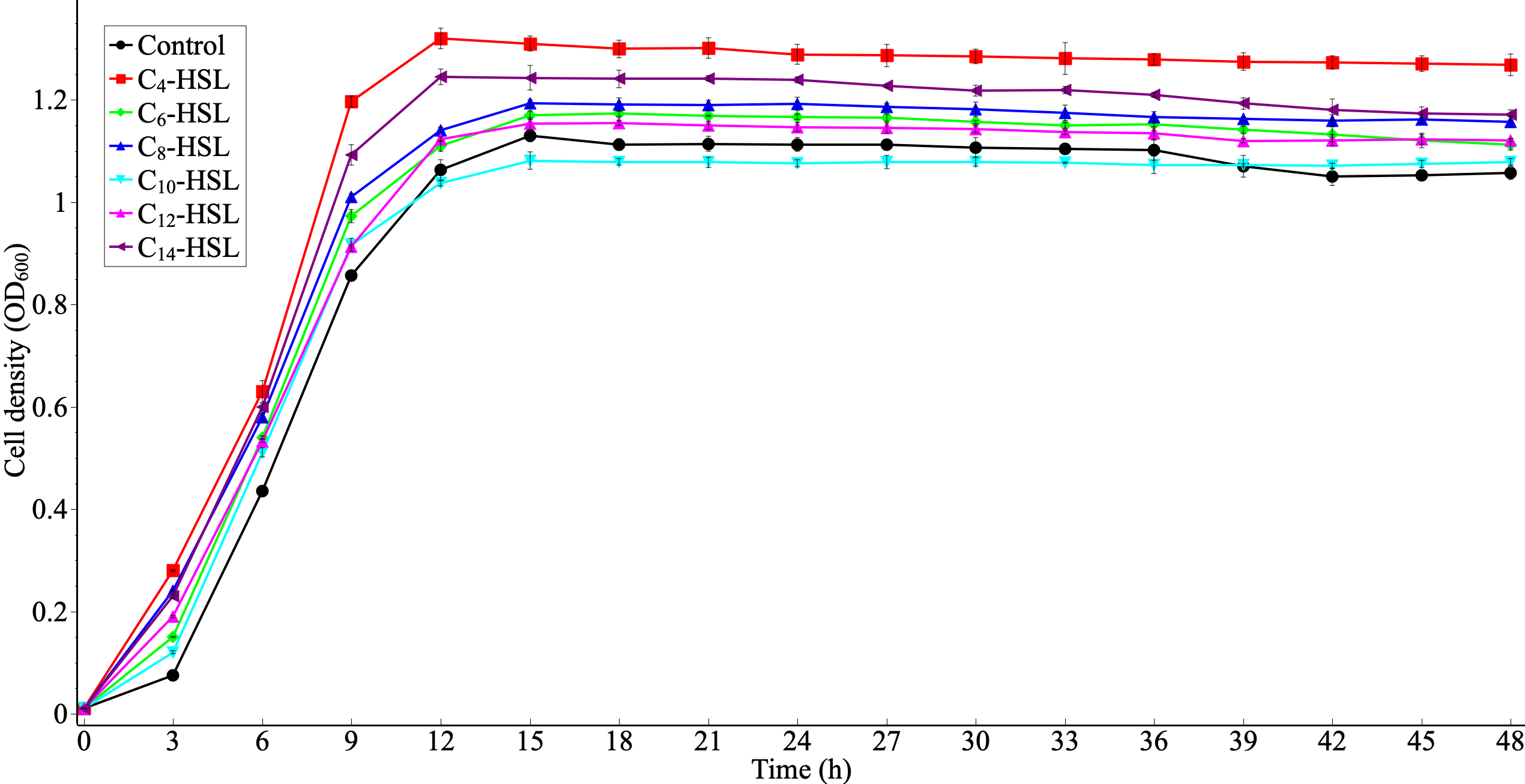


Fig. S2. GC-MS ion m/z 143 chromatograms of a mixture of standard AHL molecules (A) and the extracted supernatants of a pure *P. fluorescens* culture (B). The peaks detected at the retention time of 4.406 and 10.678 min were found to be C_4_-HSL and C_10_-HSL.


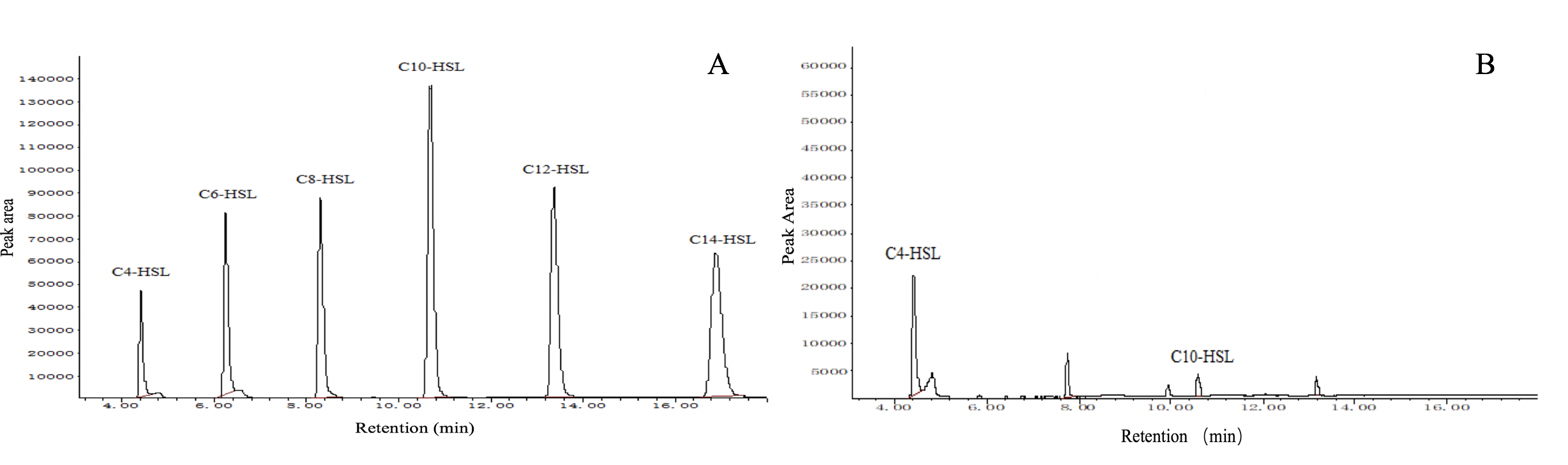


Fig. S3. Part of the sequence alignment results for RhlR proteins in *P. fluorescens* and its matching proteins.


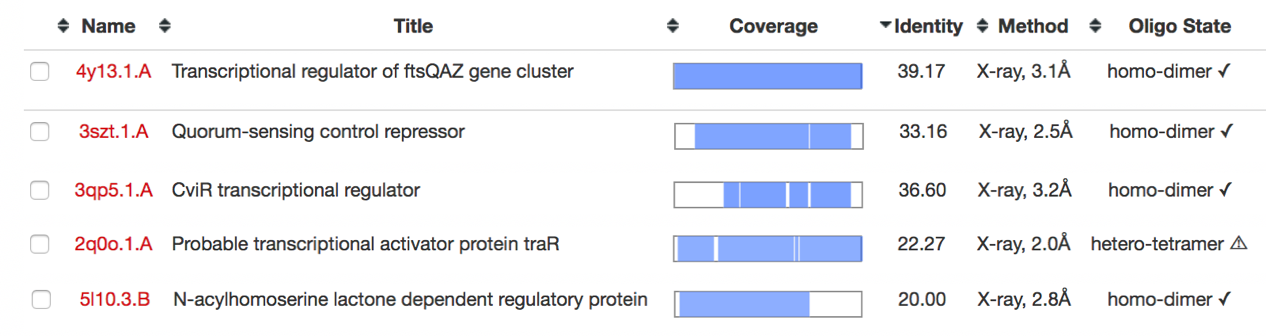


Fig. S4. Assessment of the quality of RhlR protein model of *P. fluorescens.*


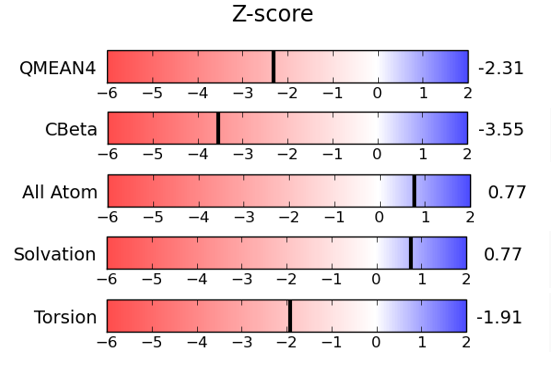

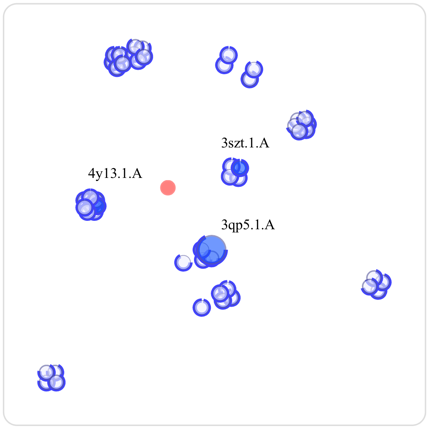

Supplement: Supplementary file 1 [file Data_Sheet_1.docx]
